# VIP Promotes Recruitment of Tregs to the Uterine–Placental Interface During the Peri-Implantation Period to Sustain a Tolerogenic Microenvironment

**DOI:** 10.3389/fimmu.2019.02907

**Published:** 2020-01-08

**Authors:** Lucila Gallino, Vanesa Hauk, Laura Fernández, Elizabeth Soczewski, Soledad Gori, Esteban Grasso, Guillermina Calo, Nora Saraco, Esperanza Berensztein, James A. Waschek, Claudia Pérez Leirós, Rosanna Ramhorst

**Affiliations:** ^1^CONICET, Laboratorio de Inmunofarmacología, Instituto de Química Biológica de la Facultad de Ciencias Exactas y Naturales (IQUIBICEN), Universidad de Buenos Aires, Buenos Aires, Argentina; ^2^Servicio de Endocrinología, Hospital Pediátrico Dr. Juan P. Garrahan, CONICET, Universidad de Buenos Aires, Buenos Aires, Argentina; ^3^Department of Psychiatry and Biobehavioral Sciences, The David Geffen School of Medicine, Semel Institute for Neuroscience and Human Behavior, University of California, Los Angeles, Los Angeles, CA, United States

**Keywords:** early pregnancy, tolerance, VIP, tregs, implantation

## Abstract

Uterine receptivity and embryo implantation are two main processes that need a finely regulated balance between pro-inflammatory and tolerogenic mediators to allow a successful pregnancy. The neuroimmune peptide vasoactive intestinal peptide (VIP) is a key regulator, and it is involved in the induction of regulatory T cells (Tregs), which are crucial in both processes. Here, we analyzed the ability of endogenous and exogenous VIP to sustain a tolerogenic microenvironment during the peri-implantation period, particularly focusing on Treg recruitment. Wild-type (WT) and VIP-deficient mice [heterozygous (HT, +/−), knockout (KO, −/−)], and FOXP3-knock-in-GFP mice either pregnant or in estrus were used. During the day of estrus, we found significant histological differences between the uterus of WT mice vs. VIP-deficient mice, with the latter exhibiting undetectable levels of FOXP3 expression, decreased expression of interleukin (IL)-10, and vascular endothelial growth factor (VEGF)c, and increased gene expression of the Th17 proinflammatory transcription factor RORγt. To study the implantation window, we mated WT and VIP (+/−) females with WT males and observed altered FOXP3, VEGFc, IL-10, and transforming growth factor (TGF)β gene expression at the implantation sites at day 5.5 (d5.5), demonstrating a more inflammatory environment in VIP (+/−) vs. VIP (+/+) females. A similar molecular profile was observed at implantation sites of WT × WT mice treated with VIP antagonist at d3.5. We then examined the ability GFP-sorted CD4+ cells from FOXP3-GFP females to migrate toward conditioned media (CM) obtained from d5.5 implantation sites cultured in the absence/presence of VIP or VIP antagonist. VIP treatment increased CD4+FOXP3+ and decreased CD4+ total cell migration towards implantation sites, and VIP antagonist prevented these effects. Finally, we performed adoptive cell transfer of Tregs (sorted from FOXP3-GFP females) in VIP-deficient-mice, and we observed that FOXP3-GFP cells were mainly recruited into the uterus/implantation sites compared to all other tested tissues. In addition, after Treg transfer, we found an increase in IL-10 expression and VEGFc in HT females and allowed embryo implantation in KO females. In conclusion, VIP contributes to a local tolerogenic response necessary for successful pregnancy, preventing the development of a hostile uterine microenvironment for implantation by the selective recruitment of Tregs during the peri-implantation period.

## Introduction

The peri-implantation period requires a variety of cellular processes that are encompassed to ensure proper trophoblast growth and invasion with intense vascular remodeling in an immunotolerant microenvironment (1–3).

Critical to implantation is an adequate decidual response associated with a threshold level of physiological inflammation that facilitates the generation of a receptive endometrium (4). However, within days of conception, inflammation needs to be controlled to sustain decidualization and implantation progress (5, 6). Regulatory T cells (Tregs, CD4+FOXP3+ cells) are critical for controlling inflammation in early pregnancy, establishing the receptive decidual environment necessary for placentation.

Considerable evidence from *in vivo* and *in vitro* models demonstrates that Tregs are generated throughout pregnancy and are critical for implantation even before mating (7–10). In DEREG-transgenic mice which express a diphtheria toxin (DT) receptor-enhanced green fluorescent fusion protein under the control of the FOXP3 promoter, the administration of DT resulted in lower pregnancy rates and smaller litters due to deficits in embryo implantation. Moreover, the adoptive cell transfer of Tregs (one injection prior to mating and the other at d2 of gestation) prevented the defective embryo implantation in this model (10). Moreover, Teles et al. showed that the absence of Tregs impaired implantation in both syngeneic and allogeneic matings (11). Regarding the origin of these Tregs, the evidence pointed out that natural Tregs (thymus origin) were present in the thymus and in the lymph nodes draining the uterus at early pregnancy, and that an expanded population of FOXP3+ cells was generated in the periphery (induced Tregs) at later pregnancy stages (12).

The critical role of Tregs with regard to implantation was also reinforced in CBA/J females mated with DBA/2J males, which show a high resorption rate associated with an imbalance in Tregs/Th1 cells. The adoptive Treg cell transfer from CBA/J females mated with BALB/c males elevates decidual Tregs, restoring fetal viability, but only when Tregs were transferred before embryo implantation (13), confirming that Tregs are essential in the peri-implantation period to manage the anti-inflammatory transition.

It has been extensively demonstrated that endometrial and trophoblastic cells contribute to the maintenance of immune homeostasis through soluble and contact factors during pregnancy (3, 14–17). One of the factors proposed to have an immunoregulatory role during implantation and at the early maternal–fetal interface is vasoactive intestinal peptide (VIP) (18, 19). VIP is produced by stromal cells (20), syncytium, and cytotrophoblastic cells in the first and third trimesters (21) by extravillous trophoblast (EVT) cells and decidual glandular cells (22, 23). VIP is an endogenous 28-amino acid peptide with strong anti-inflammatory and vasodilating activities by binding to the high-affinity specific VPAC1 and VPAC2 receptors (24–26). Indeed, this master key immunopeptide displays multiple target circuits at the interaction of maternal leukocytes with human trophoblast and endometrial cells, particularly inducing Tregs as well as their selective recruitment towards trophoblast cells (23, 27–29). In fact, at stages prior to embryo implantation, VIP through VPAC1 and VPAC2 receptors coupled to adenylate cyclase contributes to the decidualization program induction (30). In an *in vitro* model of decidualization, those endometrial stromal cells decidualized by VIP not only increased the expression of characteristic markers of decidualization but also conditioned dendritic cells to a tolerogenic profile (30).

Highlighting the relevance of endogenous VIP production, VIP knockout (KO) mice exhibit a wide variety of immune disorders (31–33). With regard to pregnancy, they present a disrupted estrous cycle compared with controls, with longer periods inter-gestations, and produce about half the offspring of their wild-type (WT) sisters even when mated to the same males (34). Moreover, impaired trophoblast cell function at placentation and failure of immune homeostasis maintenance were recently reported in a VIP-deficient pregnancy model (35); however, there are no data of VIP contribution to Treg recruitment and functional shaping previous to and during the peri-implantation period.

Here we explored the relevance of maternal VIP to uterine homeostasis and immune tolerance associated with embryo implantation with special focus on Tregs. To go further into the immunoregulatory mechanisms of maternal endogenous VIP, we used two approaches: VIP-deficient and FOXP3-GFP-knock-in females either in estrus or pregnant after mating with WT males. We demonstrate that VIP contributes to the selective recruitment of maternal Tregs toward the uterus and that the enrichment of Tregs by adoptive cell transfer improves uterine microenvironment, allowing a successful implantation.

## Materials and Methods

### Mouse Pregnancy Model

Mice were bred and maintained on a 12:12 h light–dark schedule in the Central Animal Care facility at the School of Sciences, University of Buenos Aires (FCEN-UBA). Female VIP (–/-), (+/−), WT (+/+) C57BL/6J, or FOXP3-IRES-GFP-knock-in (36) on a C57BL/6J background mice (3–8 months old) were paired with WT males C57BL/6J (37). Animals were checked daily for vaginal plugs and separated once mated. The day of the vaginal plug was considered day 0.5 of pregnancy. Pregnant mice were sacrificed at days 3.5, 4.5, and 5.5 of gestation or before mating at estrus day, and tissues were collected to evaluate gene expression and Treg frequency. All studies were conducted according to standard protocols and were approved by the Animal Care and Use Committee of the School of Sciences, University of Buenos Aires.

### *In vivo* VIP Antagonist Treatment

For the VIP antagonist treatment studies, FOXP3-GFP-knock-in mice were injected i.p. with VIP antagonist (Synthetic H-Lys-Pro-Arg-Arg-Pro-Tyr-Thr-Asp-Asn-Tyr-Thr-Arg-Leu-Arg-Lys-Gln-Met-Ala-Val-Lys-Lys-Tyr-Leu-Asn-Ser-Ile-Leu-Asn-NH2, BACHEM, USA) 100 nmol/mouse *via* an insulin syringe needle. In non-pregnant studies, three females were injected during proestrus and sacrificed during estrus. In pregnant studies, four pregnant mice were treated at day 3.5 of gestation and sacrificed at day 4.5 or 5.5 of gestation, and the implantation sites (IS) were collected to evaluate gene expression. All samples were compared with animals in the same conditions injected with phosphate buffer solution (PBS 1%).

### Determination of Estrous Cycles by Vaginal Lavage

WT (*n* = 7), VIP+/– (*n* = 7), and VIP-/- (*n* = 7) female mice (3–6 months old) housed under a 12:12 light–dark (LD) cycle were monitored to determine the estrous cycle using vaginal lavage at the same time of the day for 14 consecutive days. Precaution was taken during the vaginal lavage to avoid any damage by the pipette, and the lavage samples were spotted in small (~100 μl) drops onto glass microscope slides. Cell types contained within each vaginal smear were determined using light microscopy (40× power) and scored for estrous state, where proestrus is indicated by the predominance of nucleated epithelial cells, estrus is denoted by the absence of nucleated cells and the presence of cornified squamous epithelial cells, and lavages from mice in metaestrus and diestrus contain leukocytes (38).

### Tissue Collection

To obtain uterine tissue, mesenteric lymph nodes (MLN), inguinal lymph nodes (ILN), Peyer's patches (PP), thymus, and spleen, mice were euthanized by CO_2_ gas with confirmation by cervical dislocation. The skin abdomen was opened, ILN were exposed from each side of the animal fat tissue, and then retired and placed in 5 ml of media Dulbecco's modified Eagle medium (DMEM F12) supplemented with 10% fetal bovine serum (FBS). Then the abdomen was opened; the organs were identified, dissected out, and placed in 10 ml of fresh media. Excess fat and connective tissue were removed. Each tissue was transected into four to five donut-shaped pieces and then minced using a cell strainer [100 μm (BIOFIL)]. The cell suspension was centrifuged, the supernatant was discarded, and the resultant cell pellet was taken for cell staining or sorting.

### Flow Cytometry Analysis

Flow cytometry analysis for CD4 was performed according to the manufacturer's instruction. Briefly, 1 × 10^6^ cells were stained with anti-CD4 PE-conjugated (Becton Dickinson, San José, CA, United States). After 30 min, cells were washed, and 20,000 events were acquired in a fluorescence-activated cell sorting (FACS) Aria II cytometer® (Becton Dickinson, San José, CA, United States). The results were analyzed using the FLOWJO 7.6.2 software. Negative control samples were incubated in parallel with an irrelevant, isotype-matched Ab. Results for positive cells were expressed as a percentage of the respective population, and the quadrant was set using irrelevant isotype-specific Ab. Particularly for Treg analysis, FOXP3-positive cells were determined inside the electronically gated CD4-positive cell population.

### Sorting of GFP FOXP3+ Cells

Cells obtained from a GFP-knock-in mice as above described were sorted using the FACS Aria II cytometer® (Becton Dickinson, San José, CA, United States) as the cell population that expresses the GFP marker.

### RT-PCR

Determination of GAPDH, FOXP3, interleukin (IL)-10, RORγt, transforming growth factor (TGF)β, vascular endothelial growth factor (VEGF)c expression levels was performed in uterine tissue at different time points and immune-related organs. Briefly, total RNA was isolated following manufacturer recommendations with Trizol reagent (Life Technologies, Grand Island, NY, USA), and cDNAs were generated from 1 μg of RNA using MMLV reverse transcriptase, RNAsin, RNAse inhibitor, and oligodT kit (Promega Corporation, Madison, WI, USA) and stored at −20°C for batch analysis. The sample volume was increased to 25 μl with the solution containing 50 mM KCl; 10 mM Tris (pH 8.3); 1.5 mM MgCl2; 0.1 mM forward and reverse primers of FOXP3, IL-10, RORγt, TGFβ, VEGFc, and GAPDH as internal control (described in Table 1) and 1 U Taq polymerase in a DNA Thermocycler (PerkinElmer/Cetus, Boston, MA, United States). PCR products were electrophoresed through a 2% ethidium bromide-stained agarose gel, visualized by transillumination and scanned. Densitometry was performed using ImageJ 1.47 software (NIH, USA), and results were expressed as arbitrary units normalized to GAPDH expression.

**Table 1:** Primers used in RT-PCR assays.

**Gene**	**Forward**	**Reverse**
GAPDH	TGATGACATCAAGAAGGTGGTGAAG	TCCTTGGAGGCCATGTAGGCCAT
TGFβ	GACTCTCCACCTGCAAGACCA	TTGGGGGACTGGCGAGCCTT
FOXP3	GGCCCTTCTCCAGGACAGA	GCTGATCATGGCTGGGTTGT
RORγt	CACGGCCCTGGTTCTCAT	CAGATGTTCCACTCTCCTCTTCTCT
IL-10	GTTGCCAAGCCTTATCGGAAATG	CACTCTTCACCTGCTCCACTG
VEGFc	GATGTGGGGAAGGAGTTTGG	GATGTGGGGAAGGAGTTTGG

### Transwell Migration Assays

Conditioned media (CM) used in migration assays were obtained after 24 h of culturing implantation sites of WT or VIP+/– female mice of d5.5 with or without VIP (100 nM) or VIP antagonist (50 and 100 nM, Synthetic H-Lys-Pro-Arg-Arg-Pro-Tyr-Thr-Asp-Asn-Tyr-Thr-Arg-Leu-Arg-Lys-Gln-Met-Ala-Val-Lys-Lys-Tyr-Leu-Asn-Ser-Ile-Leu-Asn-NH2, BACHEM, United States) obtained as described above in 24-well flat-bottom polystyrene plates in 400 μl of complete DMEM-F12 10% FBS. CD4-positive cells from draining lymph nodes of a FOXP3-IRES-GFP female (2 × 10^5^ cells/well) were deposited on top of a 5-μm pore insert with polycarbonate membrane (Becton Dickinson, San José, CA, United States) and left for 24 h migration toward the different CM. After 24 h of culture, cells in the lower chamber were recovered, stained, and analyzed by flow cytometry for Treg frequency as described above.

### Histological Studies

Mice uterus was removed and then fixed in 4% paraformaldehyde overnight at room temperature. The tissues were embedded in paraffin wax, sections of 4 μm were cut and placed on silanized glass slides, and hematoxylin–eosin (H&E) staining was performed.

The count of endometrial glands was carried out in H&E samples using Leica ICC50 HD, High Definition digital microscope camera. Ten slides were observed and counted from each animal and averaged. All the cross sections of the endometrial glands for each tissue sample were counted using a Greek guard pattern.

The diameter of the glands was assessed with ImageJ® where 10 pictures in 400× were analyzed and averaged for each animal.

### Adoptive Cell Transfer of Tregs

VIP+/− female mice were continuously mated with WT males after reaching 2 months of age. The females that did not get pregnant in 6 months received an injection of Tregs and where then mated again with WT males 3 months old in the afternoon of the injection. To follow Treg *in vivo* migration, we injected 200,000 Tregs taken from the draining lymph nodes of a FOXP3-GFP-knock-in female obtained by sorting as described before. The purified Tregs were washed twice with PBS and then resuspended in 0.2 ml of PBS and injected through the tail vein. One week after, we found the vaginal plug; females were sacrificed; uterus, GI, GM, PP, and thymus were pulverized through a 100-um cell strainer, stained for anti CD4, and then analyzed by flow cytometry. Data are shown as % of GFP cells from the CD4+ population or as % of GFP+ cells from the 200,000 Treg cells injected.

In another set of experiments, 2 months of age VIP−/− females were mated during 2 weeks with 3 months old WT males and after that time, separated 2 more weeks to determine which females did not get pregnant. Those females received an injection of 200,000 Tregs taken from the draining lymph nodes of a FOXP3-GFP-knock-in female obtained by sorting as described before and mated again with the same male. Vaginal plug was checked every day and when found animals were sacrificed al d5.5.

### Statistical Analysis

The significance of the results was analyzed using Student's *t* test and Mann-Whitney test for non-parametric samples using the GraphPad Prism7 software (GraphPad, San Diego, CA, United States). When more than two comparisons were needed, we used ANOVA test and Dunn's multiple comparison posttest. A value of *p* < 0.05 was considered significant.

## Results

### VIP-Deficient Mice Show a Hostile Microenvironment in Estrus Uterus Associated With a Reduced FOXP3 Gene Expression

**Figure 1:**
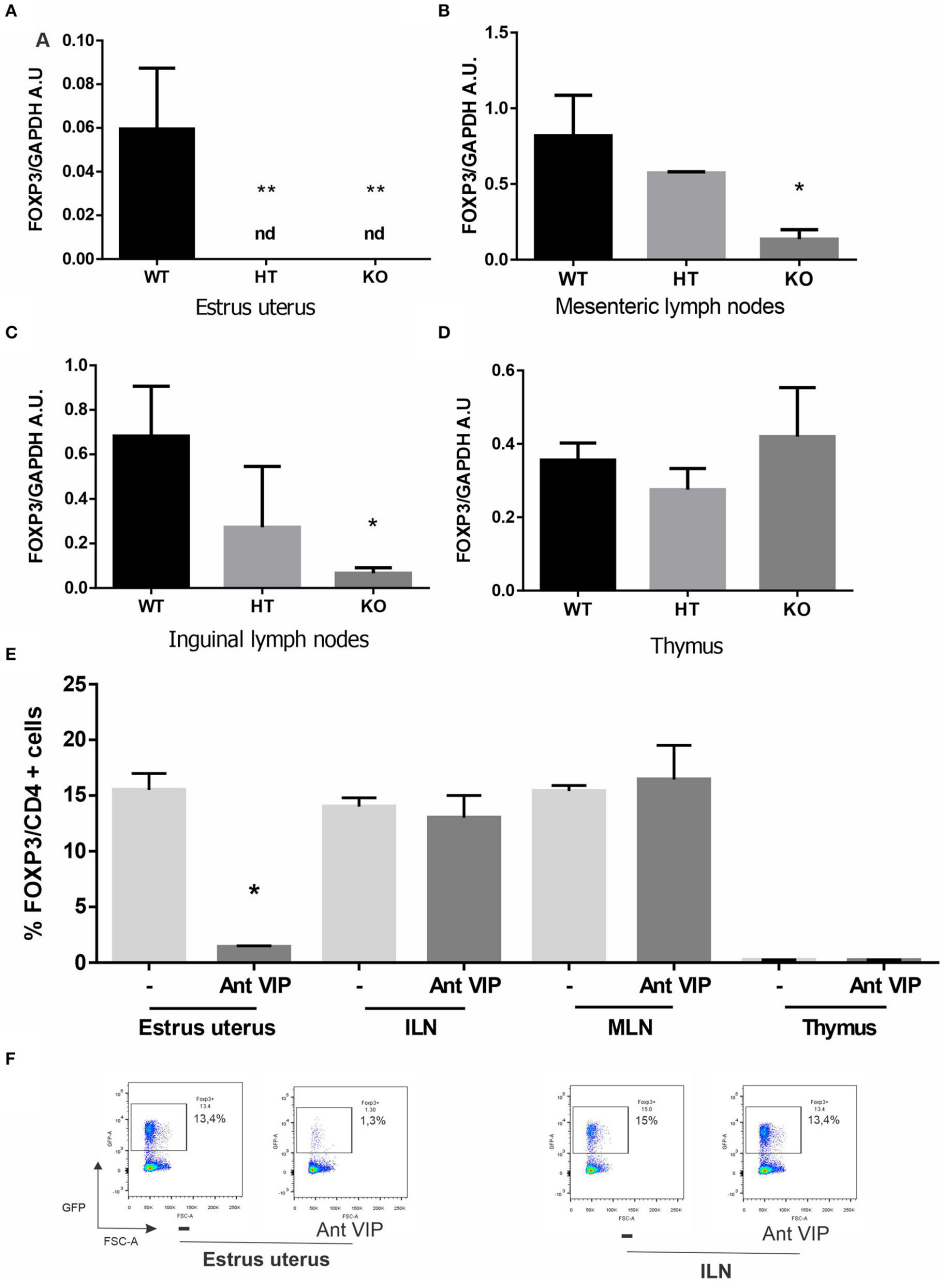
Vasoactive intestinal peptide (VIP)-deficient mice show a reduction of FOXP3 expression, and VIP antagonist treatment decreases regulatory T cell (Treg) migration in wild-type (WT) estrus uterus. The estrous cycle was evaluated in VIP−/−, VIP +/–, VIP+/+, and FOXP3-GFP-knock-in females using vaginal lavage. At estrus, VIP−/−, VIP +/–, VIP+/+ mice were sacrificed, and tissues were removed for RT-PCR analysis or fluorescence-activated cell sorting (FACS). **(A)** FOXP3 expression in uterus, **(B)** mesenteric lymph nodes (MLN), **(C)** inguinal lymph nodes (ILN), and **(D)** thymus. Bands were semiquantified with ImageJ®, and intensity was expressed in arbitrary units (AU) relative to GAPDH. Values represent mean ± SEM of at least seven experiments (ANOVA test, Dunn's post test *p < 0.05). FOXP3-GFP-knock-in females were injected during proestrus and sacrificed during estrus where the frequency of CD4+FOXP3+ was evaluated by flow cytometry analysis in uterus, MLN, ILN, and thymus **(E)**. Graphics show the % of CD4+FOXP3+ cells, and the analysis was performed inside the electronically gated CD4+ cells. Negative control samples were incubated in parallel with an irrelevant, isotype-matched Ab and used for cut-off setting. **(F)** shows representative dot plots with the % of FOXP3 cells (inside the electronically gated CD4+ cells) in the estrus uterus and ILN (Mann-Whitney test *p < 0.05 and **p < 0.01).

Female reproduction is greatly reduced in VIP (−/−) mice, with the latter producing half the offspring compared to their sisters held under the same conditions (34). Considering the relevance of Tregs to sustain a tolerogenic microenvironment during the implantation period, we evaluated FOXP3 gene expression at estrus in the uterus and other relevant tissues, such as MLN and ILN. In contrast to what was found in WT mice, FOXP3 expression was below detectable levels in VIP−/− or VIP+/− uteri at estrus (Figure 1A). Moreover, FOXP3 expression was reduced in VIP−/− (but not VIP+/−) females in MLN, ILN compared with WT, while no differences in expression were found in the thymus (Figures 1B–D). To further confirm the specific decrease of Tregs in estrus uterus, we used FOXP3-IRES-GFP females injected during proestrus with VIP antagonist to mimic KO females. We found that VIP antagonist significantly prevents the migration of FOXP3+ cells toward the estrus uterus, whereas no differences were found in MLN, ILN, nor thymus (Figures 1E,F).

**Figure 2:**
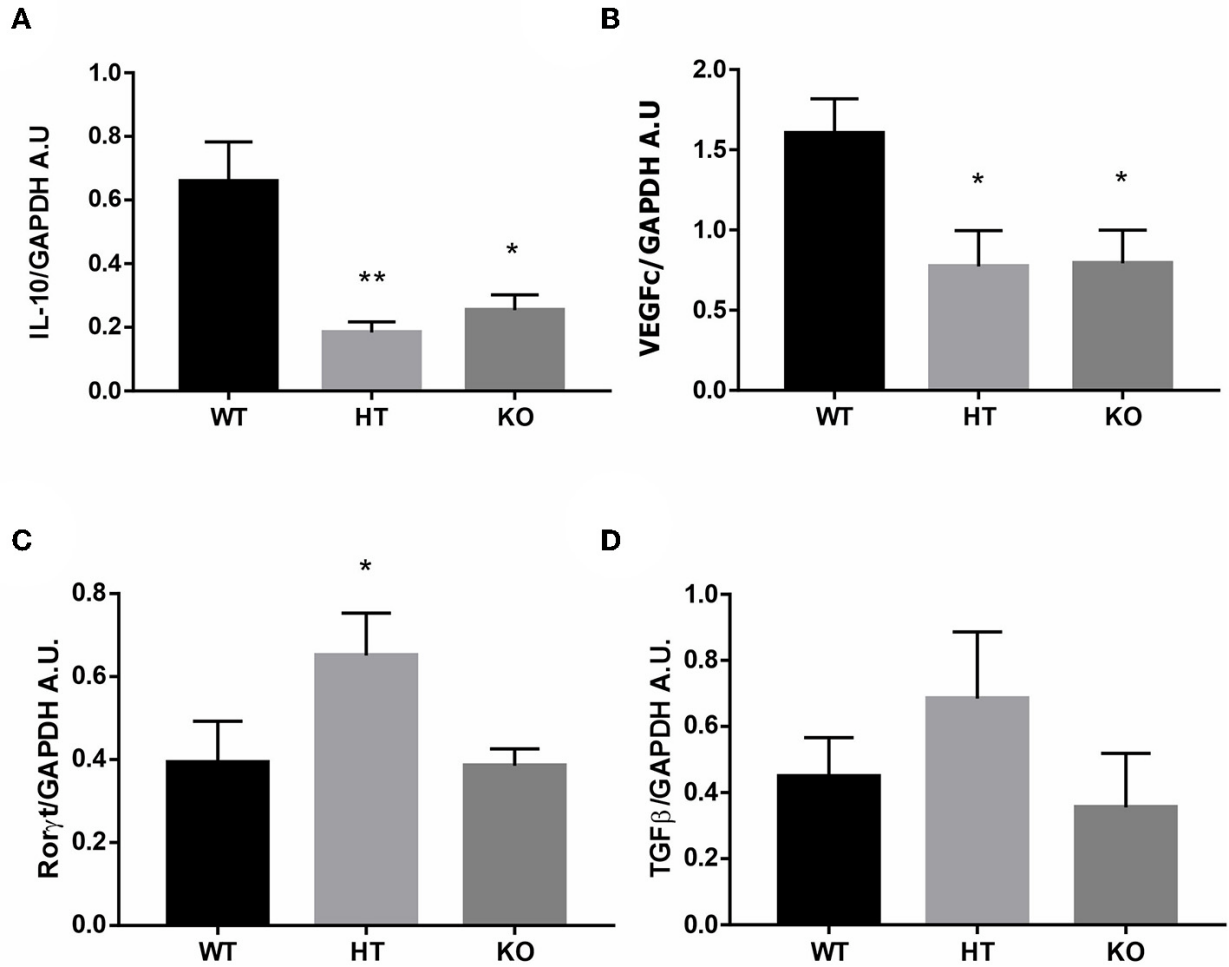
Vasoactive intestinal peptide (VIP)-deficient mice show a hostile microenvironment in estrus uterus. The estrous cycle was evaluated in VIP−/−, VIP+/–, and VIP+/+ female using vaginal lavage. At estrus, mice were sacrificed, and uterus was removed for RT-PCR analysis. **(A)** IL-10, **(B)** VEGFc, **(C)** RORγt, and **(D)** TGFß expression. Bands were semiquantified with ImageJ®, and intensity was expressed in arbitrary units (AU) relative to GAPDH. Values represent mean ± SEM of at least seven experiments (ANOVA test, Dunn's posttest *p < 0.05 and **p < 0.01).

Then we evaluated the immune profile associated with FOXP3 reduction in the uterus by measuring mRNA levels of pro-implantatory, pro-inflammatory, and anti-inflammatory markers. Estrus uterus from VIP-/- and VIP+/- females displayed a significant reduction in IL-10 and VEGFc expression and higher levels of RORγt, whereas TGFß was not modulated (Figures 2A–D).

**Figure 3:**
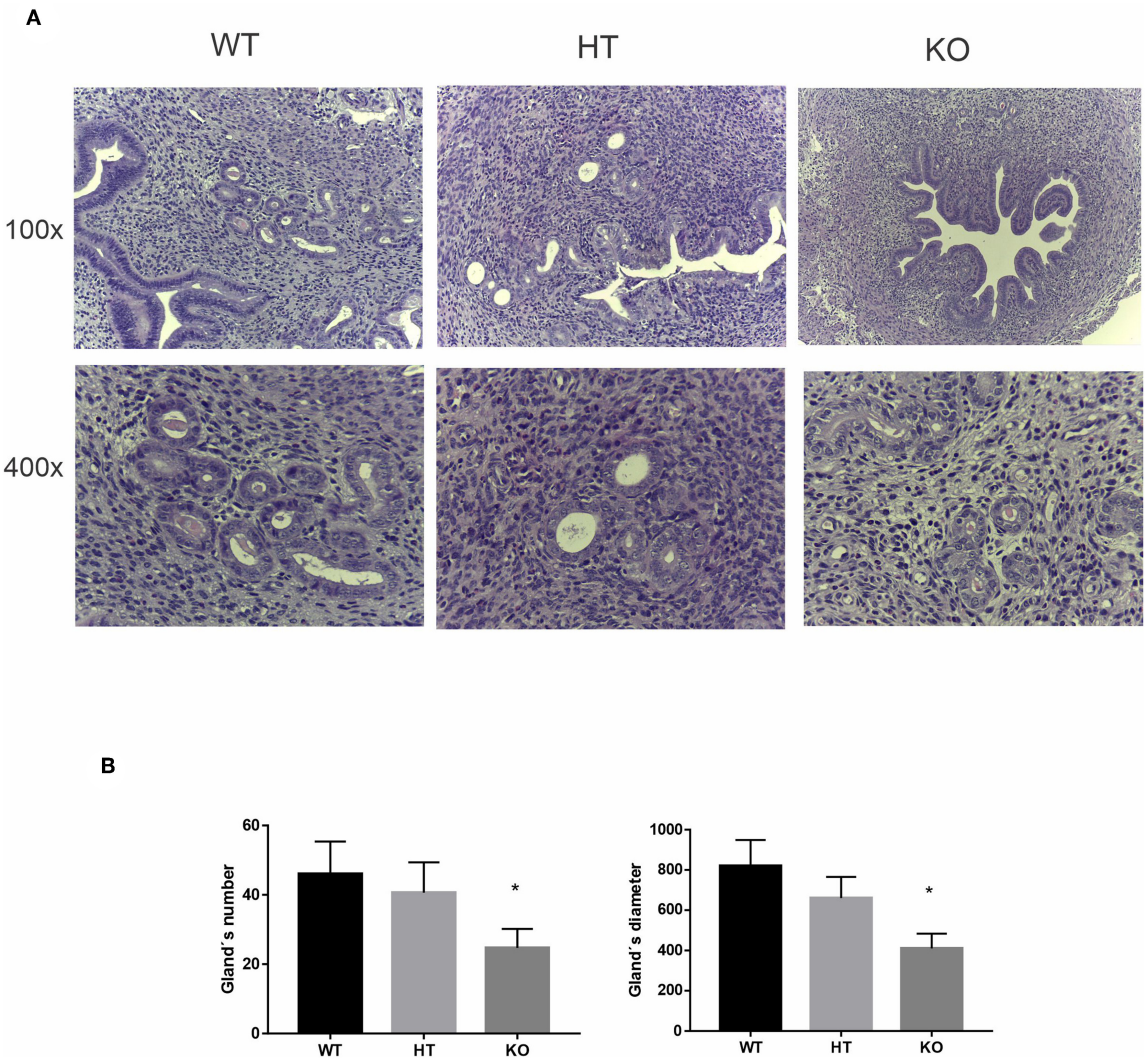
Vasoactive intestinal peptide (VIP)-deficient mice show a reduction in number and size of the uterine glands. Histologic analysis of uterine glands in estrus uterus. **(A)** Representative example of a cross section of the estrus uterus of VIP−/−, VIP+/–, and VIP+/+ in 100×, showing a detail of the glandular mesenchyme in 400× using Leica ICC50 HD, High Definition digital microscope camera. **(B)** Quantification of number and diameter of glands in 10 slides from each animal and averaged. Values represent mean ± SEM of at least three animals of each genotype (Mann-Whitney test *p < 0.05).

The uterine glands are known to be critical for uterine receptivity conditioning, decidualization, and implantation (39). Considering the active participation of the uterine glands during the peri-implantation period for the production of a wide variety of cytokines and growth factors that regulate placental cell proliferation and differentiation, and based on the trophic and prosecretory effects of VIP on exocrine glands (40), we next focused on uterine glands morphology in VIP-deficient mice. The histologic analysis of the estrus uterus revealed a marked reduction in the number of glands as well as in their diameter in VIP−/− compared with WT females, whereas VIP +/− presented an intermediate non-statistically significant decrease in number and size (Figures 3A,B).

Taken together, the present results suggest that VIP−/− and VIP+/− female mice display markers of a hostile uterine microenvironment associated with a reduction in FOXP3 expression that might interfere with nidation and embryo implantation.

### VIP Deficient Mice Show a Reduction of FOXP3 Gene Expression at the Implantation Sites

Considering that Tregs are well-known to promote a beneficial microenvironment for embryo nidation, we evaluated Tregs trafficking during early implantation toward the uterus and other regional immune tissues. First, we evaluated the CD4+FOXP3+ cell distribution in FOXP3-GFP-knock-in females mated syngenically. After the appearance of the vaginal plug, females were sacrificed, and the uterus/implantation sites, MLN, ILN, Peyer's patches (PP), spleen, and thymus were removed at 3.5, 4.5, and 5.5 days of gestation; and the Treg frequency was evaluated in each tissue by flow cytometry. Figure S1 shows that Tregs were detected in the uterus prior to (d3.5), during (d4.5), and after the day of implantation (d5.5). Moreover, when we evaluated the frequency of CD4+FOXP3+ cells in syngeneic and allogeneic pregnancies at d4.5, we did not find differences, indicating that prior to implantation, Tregs are recruited towards the uterus independently of the presence of alloantigens (Figure S2).

**Figure 4:**
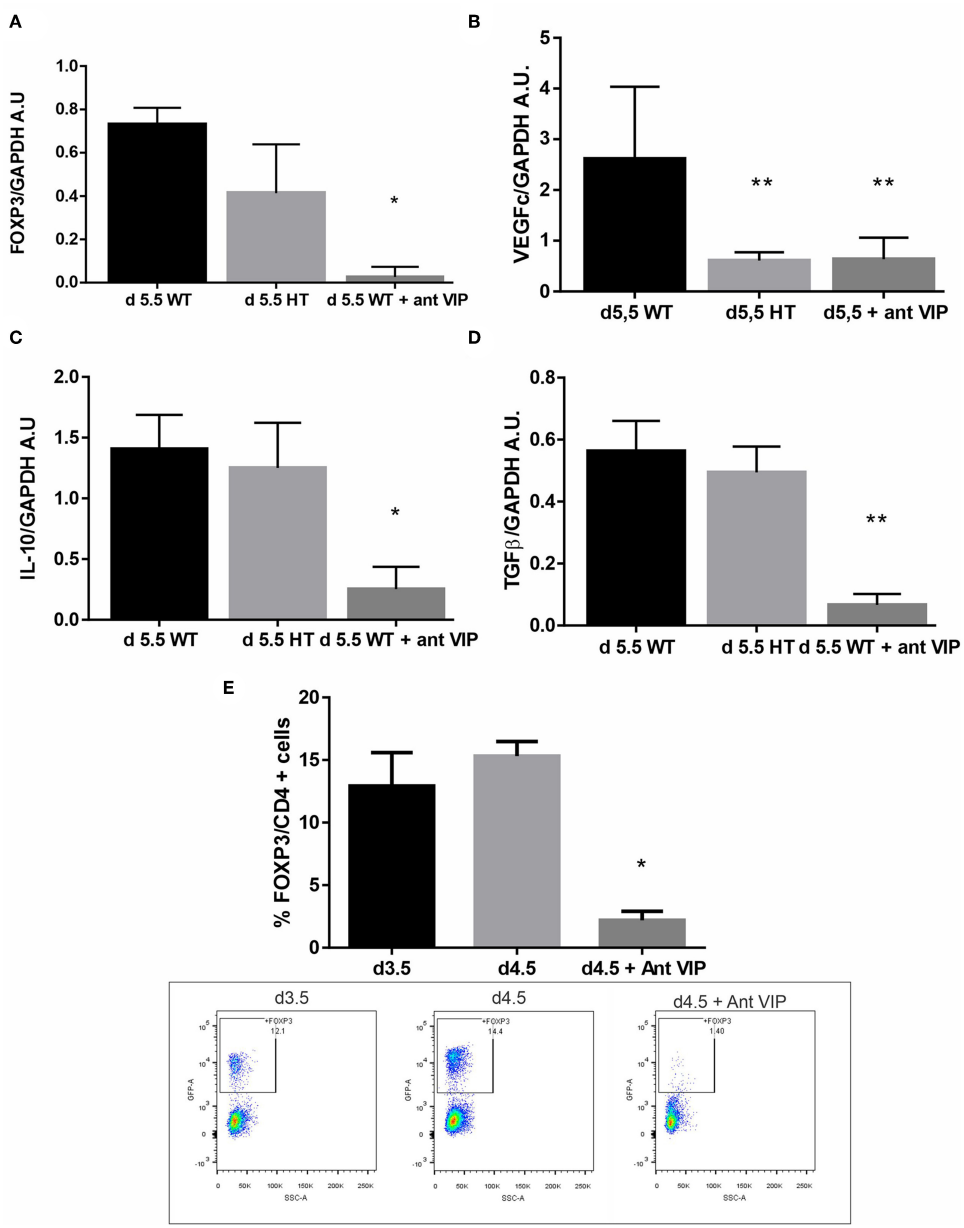
Vasoactive intestinal peptide (VIP)-deficient mice showed a reduction of FOXP3 expression at the implantation sites. VIP+/+, VIP+/−, or VIP+/− pregnant female treated with VIP antagonist at d3.5 were sacrificed at d5.5, and FOXP3 expression and other pro-implantatory markers were evaluated by RT-PCR. **(A)** FOXP3, **(B)** VEGFc **(C)**, IL-10, and **(D)** TGFβ expression. Bands were semiquantified with ImageJ®, and intensity was expressed in arbitrary units (AU) relative to GAPDH. Values represent mean ± SEM of at least three experiments (Mann-Whitney test *p < 0.05). **(E)** shows the frequency of CD4+FOXP3+ cells at the implantation sites in the peri-implantation period and the significant reduction of Treg frequency at day 4.5 caused by the injection of VIP antagonist at day 3.5. The lower panel shows representative dot plots with the % of FOXP3 cells (inside the electronically gated CD4+ cells) (Mann-Whitney test *p < 0.05 and **p < 0.01).

Then, we evaluated if VIP deficiency affects the microenvironment at the implantation sites. VIP+/− or WT females were mated with WT males, and at d5.5, they were sacrificed, and FOXP3 expression and other pro-implantatory markers were evaluated by RT-PCR. As depicted in Figure 4A, FOXP3 expression was decreased at the implantation sites at d5.5 from +/− [heterozygous (HT)] in comparison with WT sites, accompanied by a decrease in VEGFc (Figure 4B), while sustained IL-10 and TGFβ expression was observed as in the WT (Figures 4C,D).

To further confirm the specificity of the effect, we treated WT × WT pregnant female at d3.5 with a VIP antagonist (BACHEM 4031352) then sacrificed at d5.5 and analyzed for the same mediators. We observed a significant decrease in FOXP3, IL-10, VEGFc, and TGFβ at the implantation sites after *in vivo* treatment with VIP antagonist (Figures 4A–D) to levels even lower than those in HT mice. Finally, to confirm the *in vivo* effect of VIP antagonist on Tregs, we evaluated the frequency of CD4+FOXP3+ cells at the implantation sites. The injection of VIP antagonist at day 3.5 significantly reduced Treg frequency at day 4.5, suggesting that endogenous VIP normally facilitates Treg recruitment (Figure 4E, the right panel shows representative dot plots with the % of FOXP3 cells). VIP antagonist treatment during implantation does not affect Treg recruitment towards ILN, MLN, nor thymus (Figure S3).

### VIP Antagonist Treatment Prevents Treg Recruitment Towards Early Implantation Sites

**Figure 5:**
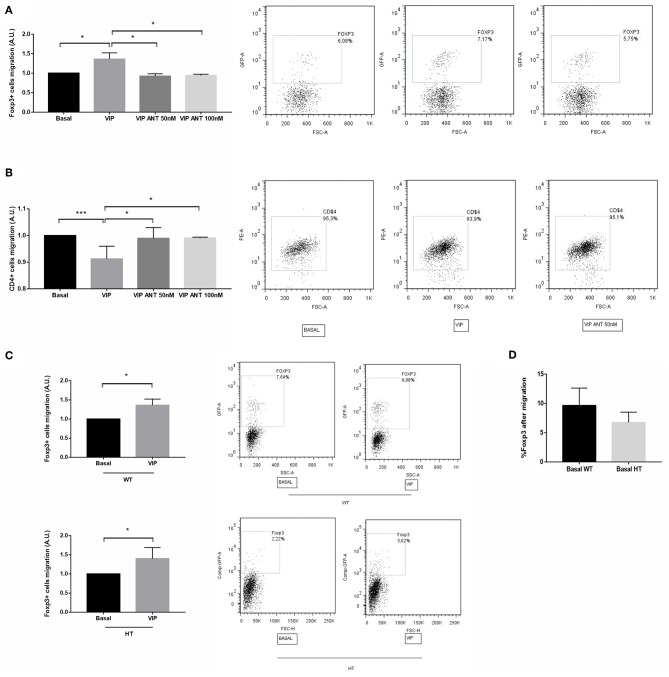
Vasoactive intestinal peptide (VIP) antagonist treatment *in vitro* prevents Treg recruitment toward early implantation site. Early implantation sites were recovered from WT × WT mating at d5.5 and treated *ex vivo* with VIP (50 nM) or VIP antagonist (50 and 100 nM) during 24 h, then the conditioned media (CM) were recovered and used for migration assays. CD4+ cells were sorted from draining lymph nodes from FOXP3-GFP-knock-in females. CD4+ cells were seeded in transwell system and set in a 24-well plate containing the CM. After 24 h, the cells were recovered from the lower compartment and the frequency of **(A)** CD4+FOXP3+ and **(B)** CD4+ cells were quantified by flow cytometry analysis. The right panel shows representative dot plots with the % of FOXP3+ cells (inside the electronically gated CD4+ cells). Negative control samples were incubated in parallel with an irrelevant, isotype-matched Ab and used for cut-off setting. **(C)** The same experiments were performed in the presence of CM from heterozygous (HT) implantation sites in the absence/presence of VIP. Comparison was performed relative to each basal migration without VIP treatment. **(D)** % of FOXP3+ cells recovered from the lower compartment after migration toward CM from WT or HT implantation site under basal conditions (without VIP treatment). Results are representative of four assays run similarly (*p < 0.05 and ***p < 0.001, Mann-Whitney Test).

Tregs are an essential population for pregnancy maintenance, and they are selectively recruited during the peri-implantation period. We thus evaluated VIP relevance in Treg recruitment toward the implantation sites. Early implantation sites were recovered from WT × WT mating at d5.5 and treated *ex vivo* with VIP (50 nM) or VIP antagonist (50 and 100 nM) for 24 h. The CM was recovered and used for migration assays. In parallel, CD4+ cells were sorted from draining lymph nodes from WT female mice and seeded in transwell systems and set in a 24-well plate containing the CM as previously described. After 24 h, the cells were recovered from the lower compartment and analyzed by FACS. We observed that the frequency of FOXP3+ cells was significantly increased when implantation sites were cultured with VIP, whereas it was decreased in the presence of VIP antagonist at both tested concentrations (Figure 5A). On the other hand, we found a decrease in the recruitment of total CD4+ cells toward CM from d5.5 treated with VIP, and this effect was prevented with VIP antagonist (Figure 5B). When we performed the same experiments in the presence of CM from implantation sites (IS) obtained from HT females at the same day, we observed that VIP treatment also significantly increased FOXP3+ cell recruitment (Figure 5C). However, CM from IS-HT induced lower migration than did that CM of WT, in accordance with *in vivo* results (Figure 5D).

We also evaluated if the treatment with VIP antagonist was able to increase the expression of RORγt. For that purpose, IS were recovered from WT × WT mating at d5.5 and treated *ex vivo* with VIP (50 nM) or VIP antagonist (2, 50, and 100 nM) during 24 h, and then RORγt was analyzed by RT-PCR. As depicted in Figure S4, VIP antagonist treatment induced a higher expression of RORγt at implantation sites in a concentration-dependent manner.

Taking together, these *in vitro* results suggest that VIP generally restrains the recruitment of the overall population of CD4 T cells, while selectively promoting the recruitment of Tregs towards the implantation site, contributing to sustain a tolerogenic microenvironment.

### The Adoptive Transfer of Tregs Improves Uterine Microenvironment in VIP-Deficient Mice and Allows Pregnancy in VIP KO Females

**Figure 6:**
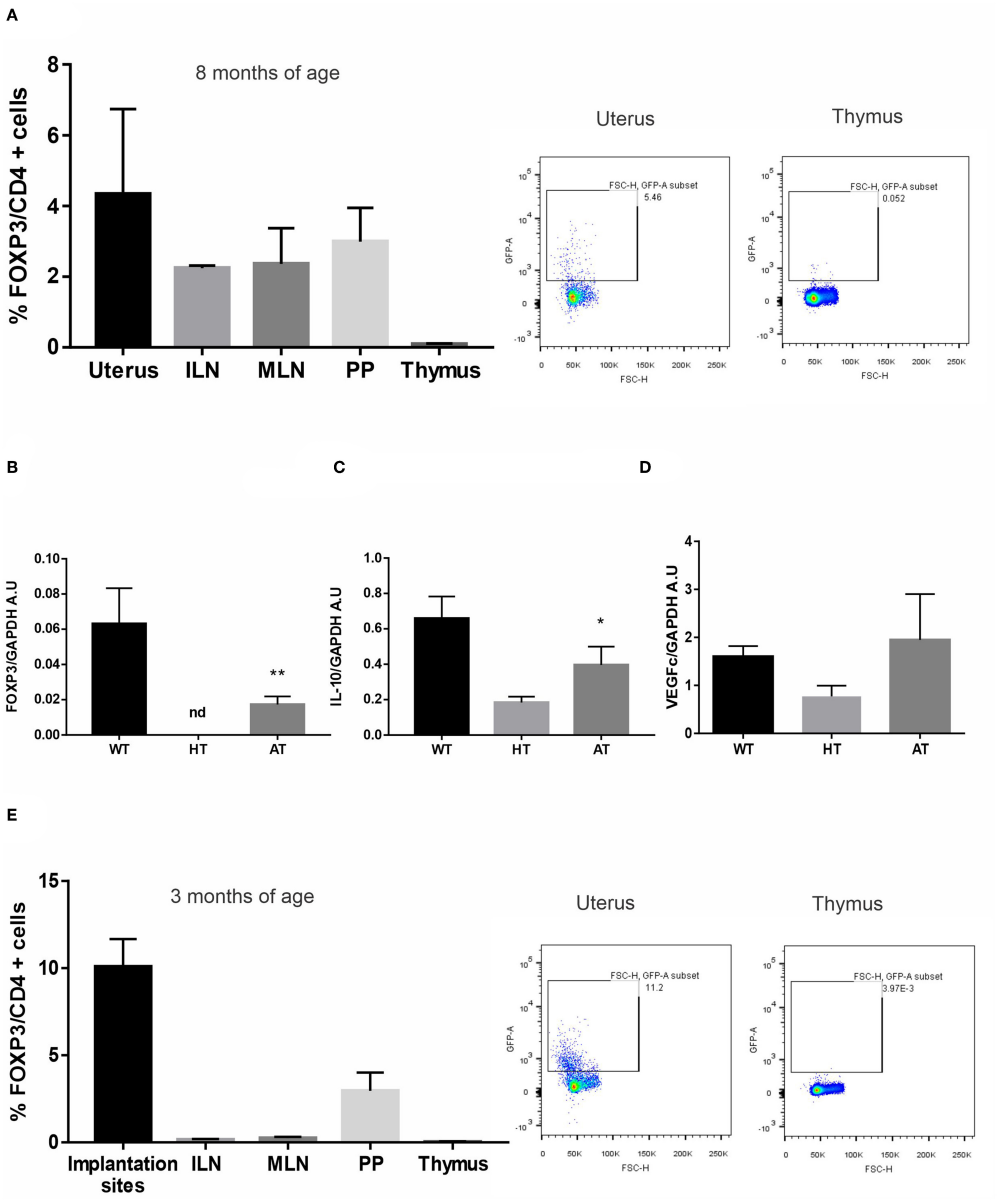
The adoptive transfer (AT)of regulatory T cells (Tregs) improves implantation site microenvironment in vasoactive intestinal peptide (VIP)-deficient mice. Tregs, FOXP3-GFP cells, were sorted from inguinal (ILN) and mesenteric lymph nodes (MLN) from FOXP3-GFP-knock-in females and were transferred to VIP heterozygous (HT) that have not gotten pregnant in 6 months. Then, they were mated with WT males, and after vaginal plug was observed, they were sacrificed at d5.5. **(A)** CD4+FOXP3+ cells frequency in uterus, ILN, MLN, Peyer patches (PP), and thymus. Results are expressed as mean % CD4FOXP3+ cells ± SEM of at least six females. The right panel figure shows representative dot plots and the frequency of FOXP3+ cells (inside the electronically gated CD4+) after AT in uterus and thymus. Negative control samples were incubated in parallel with an irrelevant, isotype-matched Ab and used for cut-off setting. **(B)** FOXP3-GFP expression in WT, HT, and after AT **(C)** interleukin (IL)-10, and **(D)** vascular endothelial growth factor (VEGF)c expression by RT-PCR. Bands were semiquantified with ImageJ, and intensity was expressed in arbitrary units (AU) relative to GAPDH. Values represent mean ± SEM of at least three experiments (Mann–Whitney test *p < 0.05). **(E)** Tregs, FOXP3-GFP cells, were sorted from ILN and MLN from FOXP3-GFP-knock-in females and were transferred to VIP KO that have not gotten pregnant for 2 weeks. Then, they were mated with WT males, and after vaginal plug was observed, they were sacrificed at d5.5. CD4+FOXP3+ cell frequency in implantation sites, ILN, MLN, Peyer patches (PP), and thymus. Results are expressed as mean % CD4+FOXP3+ cells ± SEM of at least three females. The right panel shows representative dot plots and the frequency of FOXP3+ cells (inside the electronically gated CD4+) after AT in the implantation sites and thymus. **p < 0.01.

Finally, we evaluated if the adoptive cell transfer (AT) of Tregs could improve the selective recruitment of Tregs towards the uterus of VIP-deficient females. Tregs, FOXP3-GFP cells, were sorted from ILN and MLN from FOXP3-GFP-knock-in females and were transferred to 8-months infertile VIP HT (+/−). Then, they were mated with WT males. After vaginal plugs were observed, females were sacrificed at d5.5. As depicted in Figure 6A, FOXP3-GFP cells were mainly recruited to the uterus compared to all other tested tissues (absolute numbers of FOXP3 + cells are depicted in Figure S5). In fact, not only the frequency of Tregs was increased after AT, but also IL-10 and VEGFc expression (Figures 6B–D).

In another set of experiments, Tregs, FOXP3-GFP cells, were transferred to 3 months of age VIP KO(−/−) that did not get pregnant in 2 weeks. After vaginal plugs were observed, females were sacrificed at d5.5. We observed that two out of three females (66%) that received Tregs got pregnant, whereas the pregnancy rate in KO females that did not receive the Tregs transfer was four out of 15 females (26%). In addition, FOXP3-GFP cells were mainly recruited to the implantation sites compared to all other tested tissues (Figure 6E).

Taken together these results suggest that the adoptive transfer of Tregs might improve the immune microenvironment to sustain pregnancy.

## Discussion

The basis for our studies is that (1) the initial pro-inflammatory response characteristic of embryo implantation is actively modulated to a predominant anti-inflammatory and tolerogenic profile at early gestation, (2) Tregs are essential for this immune switch, (3) VIP displays anti-inflammatory and tolerogenic effects, and that (4) VIP-deficient mice display pregnancy deficits. We thus analyzed the role of this immunopeptide as an endogenous local regulator of the maintenance of the immune homeostasis at the uterus and during the peri-implantation period, focusing on Treg recruitment.

Results presented herein provide experimental evidence that VIP contributes to the selective recruitment of maternal Tregs to the uterus, mitigating the pro-inflammatory uterine microenvironment in final preparation for implantation. Our conclusions are based on several observations. First, VIP HT and KO female displayed a molecular profile indicative of an enhanced inflammatory/hostile microenvironment for embryo nidation at estrus uterus compared to WT females, with a marked reduction of FOXP3 expression, increased expression of pro-inflammatory markers, and a reduced number and diameter of the uterine glands.

Second, after embryo implantation at d5.5, VIP-deficient mice still had a reduction in FOXP3 expression. In fact, the treatment of WT female mated with WT males with VIP antagonist at d3.5 reduced FOXP3 as well as IL-10, TGFβ, and VEGFc expression and diminished Treg recruitment, highlighting VIP relevance in the peri-implantation period. Third, *ex vivo* migration assays showed that VIP induced the selective recruitment of Tregs while restraining CD4+ cell migration. Furthermore, the VIP antagonist prevented Treg migration at two tested concentrations. Finally, the injection of FOXP3-GFP cells, obtained from ILN and MLN, to VIP-deficient females that did not got pregnant in 6 months increased the selective recruitment of FOXP3-GFP cells towards the uterus and the expression of IL-10, favoring a tolerogenic microenvironment. In contrast, adoptive transfer of Tregs to 3 months of age KO females resulted in a higher pregnancy rate compared to age-matched not injected KO females.

The immunoregulatory role of VIP at the early maternal–placental interface is based on its ability to promote an anti-inflammatory and tolerogenic microenvironment by altering the targeting of maternal leukocytes (19). For example, VIP produced by trophoblast cells induces a regulatory/suppressor macrophage phenotype or M2 profile (23, 29), affects monocytes migratory ability against pathological stimuli (41), deactivates neutrophils at the maternal–placental interface (28), and induces Tregs by a mechanism dependent of TGFβ (20, 42).

Previous to implantation, VIP production in human endometrial stromal cells (HESC) increases after 2 days of differentiation, supporting the hypothesis that VIP might contribute to the decidualization program from the earliest stages (30). Decidualization also decreased DPP4 (CD26), an intrinsic membrane glycoprotein and a serine exopeptidase expressed in glandular and endometrial stromal cells able to cleave VIP, indicating that VIP may have an increased half-life (43, 44). In fact, using an *in vitro* model of human decidualization, it was demonstrated that decidualized cells have the ability to restrain the attraction of CD4+ cells, potentially Th1, while recruit Tregs as a strategy that might prevent potential tissue damage (30).

It is important to highlight the fact that Treg cells induced in the peri-implantation phase of pregnancy are crucial for a bystander suppression throughout pregnancy (6, 45). Treg migration increases previous blastocyst stable adhesion and invasion into the uterus to exert its suppressive function at implantation, as we depicted here in the FOXP3-GFP-knock-in model (Figure S1) and in other models by others groups (7, 11). In fact, Shima et al. demonstrated that the induction of Tregs specific for paternal antigens in the uterine-draining lymph nodes just before implantation and pregnant uterus after implantation at 5.5d resulted in successful implantation and the maintenance of allogeneic pregnancy (7).

VIP contributes with Treg recruitment while it restrains CD4+ cell recruitment toward the implantation sites. The present results agree with previous reports in mice showing that effector T cells cannot accumulate within the decidua by epigenetic silencing of key T cell-attracting inflammatory chemokine genes in decidual stromal cells (46).

In fact, pregnant WT female mice carrying VIP-deficient embryos and therefore VIP-deficient trophoblasts exhibited reduced trophoblast migration and invasion capacities, accompanied by a diminished number of Treg cells at the implantation sites along with lower expression of proangiogenic and anti-inflammatory markers (35). These findings detected at the implantation sites at early stages were followed by an abnormal placental structure and lower fetal weight. This effect was overcome by VIP treatment to the early pregnant mice (35).

Considering the previous evidence, it is reasonable to infer that insufficient Tregs in the peri-implantation period will alter decidual environment and fail to achieve the appropriate inflammatory resolution that requires the invasion of trophoblasts and the maternal vessel remodeling (6). Treg abnormality in either function or number were associated with recurrent pregnancy loss (RPL) and also with recurrent implantation failure (RIF) (47, 48). It was reported that Treg migration from the peripheral blood toward the decidua decreases in cases of miscarriage with a normal or abnormal embryo karyotype (49, 50). Also an intrinsic deficiency in peripheral blood Tregs in RPL is associated with diminished IL-2 and TGF-β secretion whereas decidual Tregs have elevated interferon (IFN)-γ expression (51). Tregs that express insufficient FOXP3, due to polymorphisms in the promoter region, may be phenotypically plastic and convert into Th17 and then could directly contribute to the pathology. Moreover, reduced endometrial expression of FOXP3 indicates fewer Tregs and is associated with RIF since *in vitro* fertilization treatment success correlates with circulating levels of CD4+CD25+FOXP3+ cells (52, 53).

In this context, we have previously reported that patients with RPL displayed a significantly lower frequency of endometrial CD4+VIP+ cells in comparison with fertile women (27).

Available evidence supports the hypothesis that uterine glands and, by inference, their paracrine-acting secretions have important biological roles in blastocyst implantation, establishment of uterine receptivity, and stromal cell decidualization (39). The marked reduction in the number and size of the endometrial glands in estrus uterus from VIP KO females must contribute to a hostile microenvironment for implantation. Endometrial glands are best developed and most active during early human pregnancy and provide an important source of nutrients, growth factors. and cytokines for the feto-placental unit (54, 55). During the first trimester, the deciduochorial state confirmed the importance of these secretions for normal development of the conceptus (56, 57). In line with this, intense immunolabelling of VIP was recently demonstrated in exocrine glands of human first-trimester placenta (22).

Furthermore, we have demonstrated that adoptive transfer of Tregs to VIP-deficient females is able to improve uterus microenvironment to sustain pregnancy. It is interesting that we also observed an increase in VEGFc associated in humans with the peri-implantation period (58), and recently, it was reported that in a mouse model of Treg-cell depletion, it could cause increased inflammation and aberrant uterine artery function (59). However, whether Treg cells control the maternal vascular function is still unknown.

Advancements in assisted reproductive technologies to optimize embryo production have allowed clinicians to overcome many deficiencies in human reproduction; however, embryo implantation is still a “black box.” In *in vitro* fertilization, even the healthiest blastocyst embryo fails to implant in a normal uterus, and this fact prompts the research of novel therapeutic approaches to help couples with infertility. This represent a promising area of future research to address the so-called “endometrial factor” infertility, for which there are currently no effective treatments. Our data reveal previously unknown and useful information about VIP's contribution to uterus receptivity, embryo implantation, and peri-implantation period that might be relevant to understand infertility associated with an endometrial factor.

## Data Availability Statement

The datasets generated for this study are available on request to the corresponding author.

## Ethics Statement

The animal study was reviewed and approved by CICUAL, School of Sciences, University of Buenos Aires.

## Author Contributions

CP and RR designed the study, supervised the experimental work, wrote the manuscript, and supervised the whole study. JW provided the VIP-deficient mice. LG and VH carried out all the experiments using both mice models (*in vivo* and *ex vivo* designs). NS and EB performed the tissue analysis showed. EG, ES, SG, GC, and LF did the RT-PCRs data analysis and interpretation. All authors read and approved the final manuscript.

### Conflict of Interest

The authors declare that the research was conducted in the absence of any commercial or financial relationships that could be construed as a potential conflict of interest.
