# Supplementary material for: VIP Promotes Recruitment of Tregs to the Uterine–Placental Interface During the Peri-Implantation Period to Sustain a Tolerogenic Microenvironment
Source: Front Immunol. 2020 Jan 8;10:2907. doi: 10.3389/fimmu.2019.02907 (PMC6960177; doi:10.3389/fimmu.2019.02907)
Supplement: Supplementary file 1 [file Data_Sheet_1.pdf]

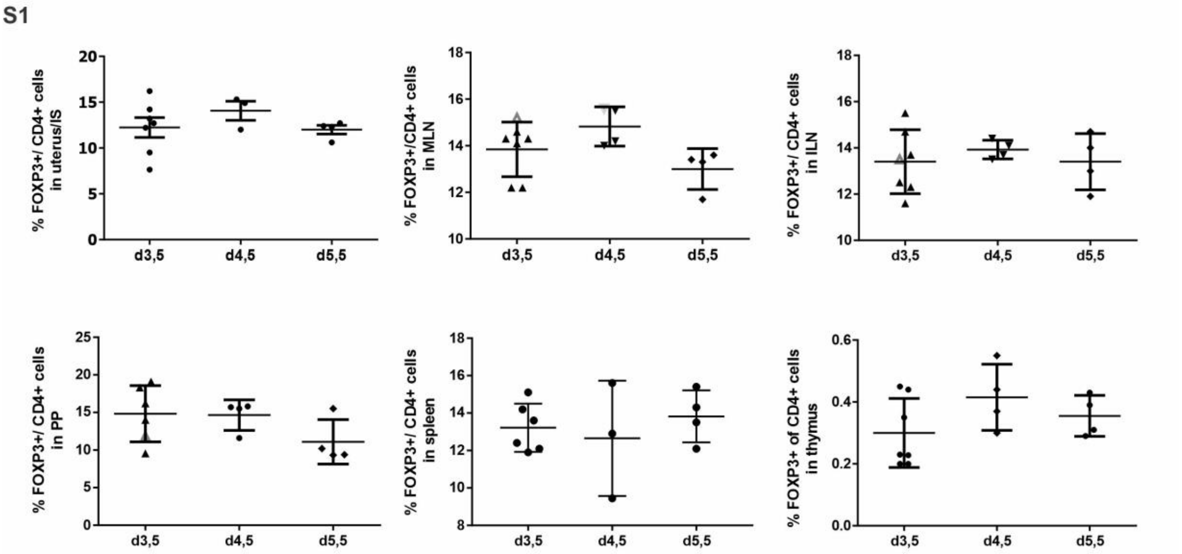

Figure S1: CD4+FOXP3+ kinetic during the peri-implantation period in FOXP3-GFP-knock in females. FOXP3-GFP-Knock-in females were mated syngenically with WT males. After the appearance of the vaginal plug females were sacrificed and the frequency of CD4+FOXP3+ was evaluated by flow cytometry analysis in uterus/implantation sites (IS), mesenteric lymph nodes (MLN), inguinal lymph node (ILN), Peyer's patches (PP), spleen, and thymus at d3.5, d4.5 and d5.5 of gestation. Graphics show the % of CD4+FOXP3+ cells and the analysis was performed inside the electronically gated CD4+ cells. Negative control samples were incubated in parallel with an irrelevant, isotype-matched Ab and used for cut off setting.

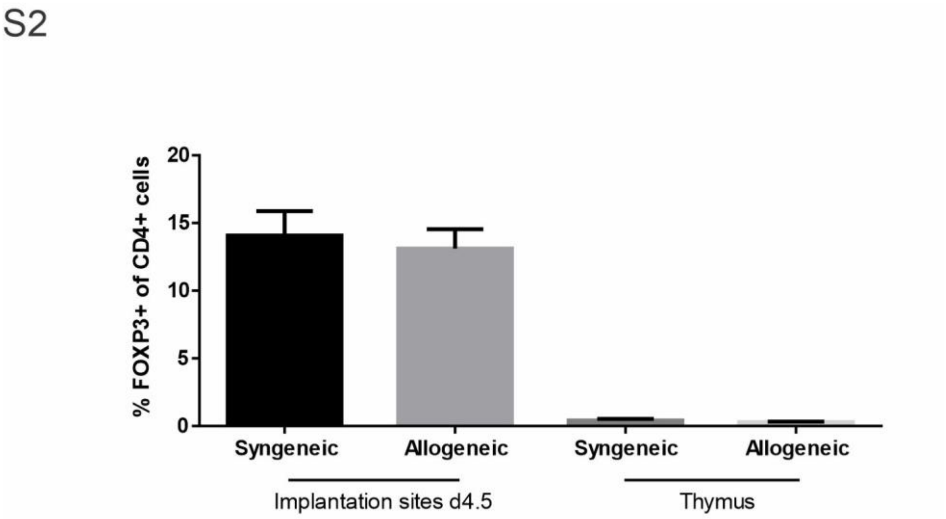

Figure S2: CD4+FOXP3+ frequency previous implantation in allogenic and singeneic pregnancy. FOXP3-GFP-Knock-in females were mated allogeneic or syngeneic with WT males. After the appearance of the vaginal plug females were sacrificed and the frequency of CD4+FOXP3+ was evaluated flow cytometry analysis at day 4.5 of gestation at implantation sites and thymus. Graphic shows the % of CD4+FOXP3+ cells and the analysis was performed inside the electronically gated CD4+ cells. Negative control samples were incubated in parallel with an irrelevant, isotype-matched Ab and used for cut off setting.

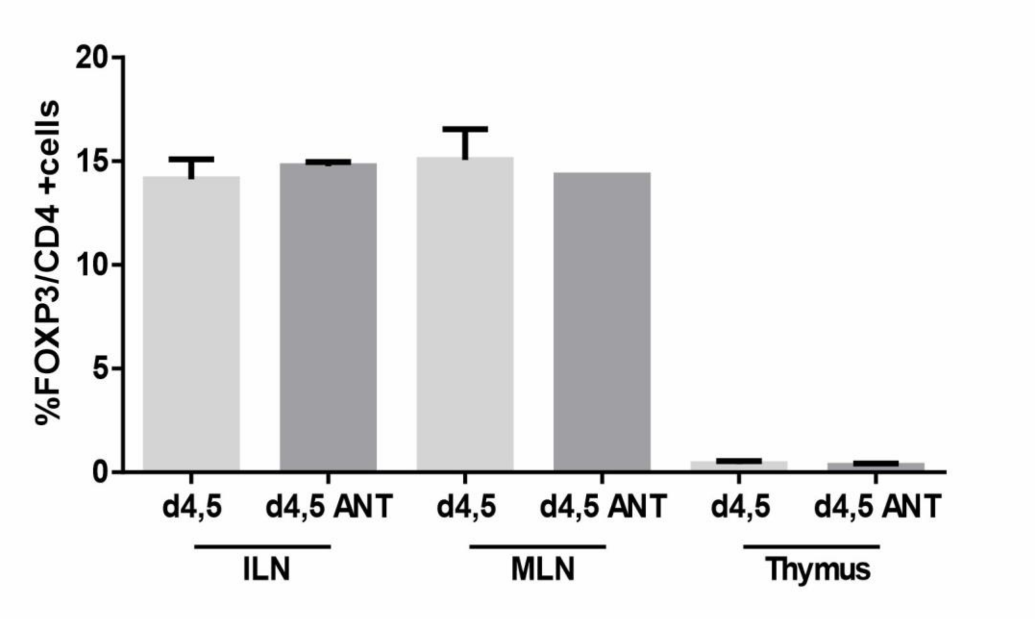

Figure S3: VIP-Antagonist treatment during implantation does not affect Tregs recruitment towards inguinal lymph nodes (ILN), mesenteric lymph nodes IMLN), nor thymus. FOXP3-GFP-Knock-in females were mated WT males. After the appearance of the vaginal plug females were treated or not with VIP-Antagonist at day 3.5, sacrificed at day 4.5 and the frequency of CD4+FOXP3+ was evaluated flow cytometry analysis at implantation sites, ILN, MLN and thymus. Graphic shows the % of CD4+FOXP3+ cells and the analysis was performed inside the electronically gated CD4+ cells. Negative control samples were incubated in parallel with an irrelevant, isotype-matched Ab and used for cut off setting.

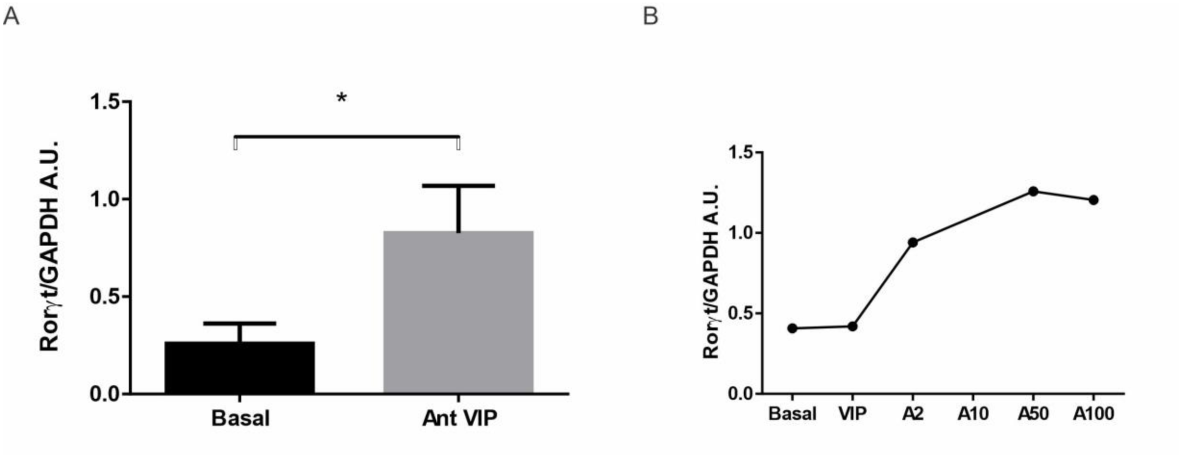

Figure S4: VIP-Antagonist *in vitro* induces a higher expression of RORγt at implantation sites. Early implantation sites were recovered from WTxWT mating at d5.5 and treated *ex vivo* with VIP (50nM) or VIP-Antagonist (2, 50 and 100nM) during 24h. After culture, implantation sites were user for RT-PCR analysis. Graphic shows **(A)** RORγt expression and **(B)** a representative example. Bands were semi-quantified with ImageJ® and intensity expressed in arbitrary units (A.U.) relative to GAPDH. Values represent mean ± S.E.M of at least 3 experiments (Mann Whitney Test *p< 0.05).

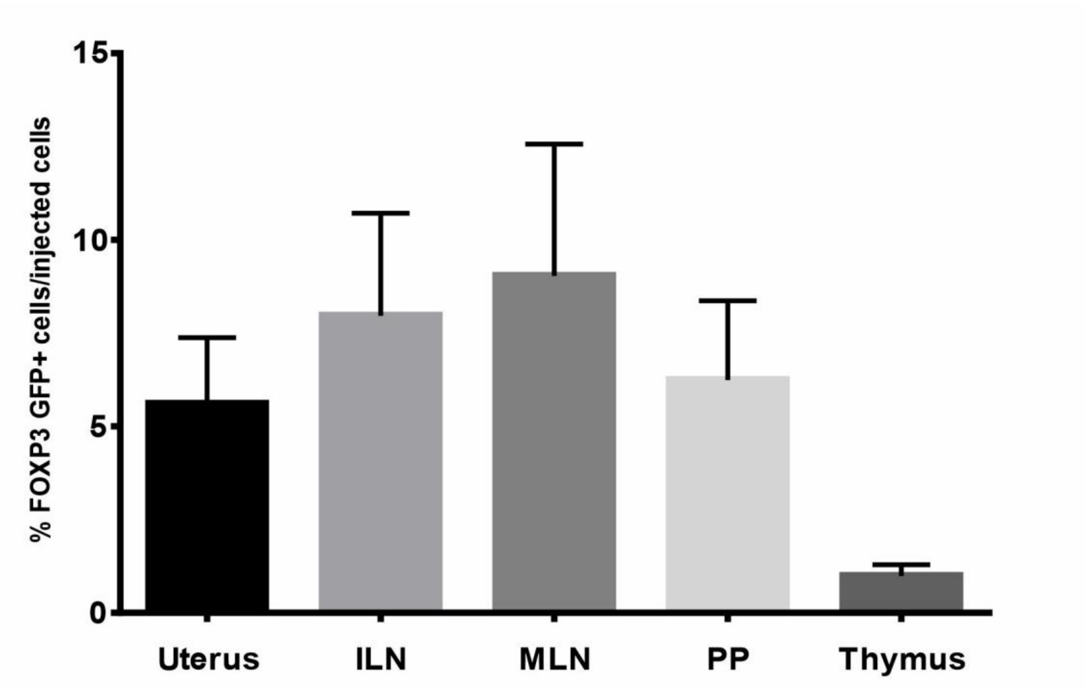

Figure S5: After the adoptive cell transfer, Tregs migrate to the uterus but also ILN, MLN, PP and Thymus. Tregs, FOXP3-GFP cells, were sorted from inguinal and mesenteric lymph nodes from FOXP3-GFP-knock-in females and were transferred to VIP HT that haven't got pregnant in 6 months. Then were mated with WT males, and after vaginal plug was observed were sacrificed at d5.5. FOXP3 GFP+ cells are expressed as total number of cells found in uterus, ILN, MLN, PP and Thymus in relation to the 200.000 cells injected. Results are expressed as mean % FOXP3+ cells/injected cells ± S.E.M. of at least 6 females. Negative control samples were incubated in parallel with an irrelevant, isotype-matched Ab and used for cut off setting.
